# Quantitative visualization of pectin distribution maps of peach fruits

**DOI:** 10.1038/s41598-017-09817-7

**Published:** 2017-08-24

**Authors:** Nan Zhu, Weinan Huang, Di Wu, Kunsong Chen, Yong He

**Affiliations:** 1 College of Agriculture & Biotechnology, Zhejiang Provincial Key Laboratory of Horticultural Plant Integrative Biology, The State Agriculture Ministry Laboratory of Horticultural Plant Growth, Development and Quality Improvement, Zhejiang University, Zijingang Campus, Hangzhou, 310058 P.R. China; 2 College of Biosystems Engineering and Food Science, Zhejiang University, Hangzhou, 310058 China

## Abstract

Pectin content is an important quality index of fruits, as pectin content undergoes significant changes during the peach ripening process. The commonly used carbazole colorimetry method measures only the total content value of each kind of pectin for each pulp sample and cannot provide distribution maps of the pectin contents for the whole fruit pulp. This work used the hyperspectral imaging technique to quantitatively visualize the distribution maps of pectin contents inside peach pulp at the pixel level. The protopectin contents were well predicted, with the best residual predictive deviation of 2.264, whereas the predictions of the water-soluble pectin and the total pectin contents were not satisfied. On the basis of the best predictive model, the distribution maps of the protopectin contents were quantitatively visualized. A histogram of an example protopectin distribution revealed the existence of a wide range of protopectin contents in peach pulp. Our results show that hyperspectral imaging holds promise as a powerful alternative to the carbazole colorimetry method for measuring the spatial variations in the protopectin distribution inside peach pulp. The distribution maps could be used as a maturity indicator to understand and evaluate the ripening process of peach fruit in depth.

## Introduction

The composition of cell walls in fruits is important to both consumers and industry^1, 2^. From the industry perspective, cell walls of fruits provide a rich resource of pectin and are considered as an important factor in influencing the effectiveness of many technological processes^3^. On the other hand, cell walls from fruits not only provide dietary fibers to consumers^4, 5^ but also determine the texture of fruits, which can influence the sensory acceptability of consumers^2, 6^. Therefore, knowledge about cell wall composition is important for understanding the changes in fruit texture properties that occur during the fruit development and maturation periods^7^.

Pectins are heterogeneous polysaccharides^1, 8–10^ mainly located in the primary cell wall and central lamella of terrestrial plants^11^. Pectins may be responsible for controlling the cell wall porosity and for inducing the binding of neighboring cells^12^. Solubilization and depolymerization of pectins were found to result in the weakening and disintegration of the cell wall^1^. Indeed, the flesh softening of most fruits is accompanied by pectin solubilization and the associated cell wall swelling^13^. Water-insoluble pectin content was considered to have an impact on the texture of peach fruits during storage^14^. Jia, *et al*.^15^ found that the softening of peach fruit is accompanied by the transition of water-insoluble pectins into water-soluble pectins during maturation. A decrease of protopectin (water-insoluble pectin) and an increase of water-soluble pectin contents occurred in ‘Okubo’ peach during storage^16^. Moreover, peach pulp is heterogeneous, pectins vary spatially inside the fruit. Therefore, the distribution maps of pectin contents for peach pulp could provide a detailed understanding the ripening process of a fruit, leading to further optimization of preharvest planting patterns and postharvest storage strategies. Knowing the spatial organization of the components or even the molecules at both the tissue and cellular levels will provide fundamental insights into plant biology^17^.

The sulfuric acid-carbazole colorimetry method is commonly used to measure the pectin contents of fruits and vegetables, such as peach^18^, carrot^19^, citrus^20^ and tomato^21^. This method involves pectin extraction from pulp tissue, pectin depolymerization, carbazole reaction and spectrophotometric measurements^21^. However, spatial information is frequently lost in the sulfuric acid-carbazole colorimetry method, as the analysis is performed on a tissue homogenate. Therefore, the colorimetry method only provides a total content value of each kind of pectin for each pulp sample and cannot provide detailed distribution maps of pectin contents of the fruits. The measured pectin contents by the colorimetry method lack spatial information, and the measurments depend on the location where the measurements are taken. Therefore, the carbazole colorimetry method is not representative for understanding the detailed pectin variance of heterogeneous peach pulp, as different parts of pulp have different pectin contents. New analytical tools are required to gain knowledge about the pectin spatial distribution for peach fruits to improve our basic knowledge of quality variation during peach fruit development and maturation as well as facilitate the advancement of peach preharvest planting patterns and postharvest storage strategies.

Imaging techniques are accessible ways instead of location based measurements to provide a detailed visualization of target quality attributes at the pixel level. Of the various non-invasive imaging methods, hyperspectral imaging (also called imaging spectroscopy or imaging spectrometry) has been reported in the assessment of the quality distribution of many fruits in the past decade^22^. Hyperspectral imaging is a spectral imaging technique that integrates both spectroscopy and computer vision techniques into one system, resulting in the capability to generate a spatial map with spectral variation. For a hyperspectral image, each pixel within the imaging plane has a complete wavelength spectrum over the whole available spectral range, which commonly consists of visible and near-infrared spectral data, so that the hyperspectral image is a three-dimensional data cube based on the combinations of the series of spectral data at each pixel, which has one spectral dimension and two spatial dimensions. Therefore, hyperspectral imaging has the capability to simultaneously quantitatively measure the inherent chemical and physical properties of the specimen as well as their spatial distribution. Currently, hyperspectral imaging has proved its capability to quantitative predict many quality attributes of fruits, such as total soluble solids (TSS) and firmness of apples^23^; TSS, moisture content and firmness of bananas^24^; mechanical properties of blueberries^25^; and TSS of kiwifruits^26^. Especially for peach, Cen, *et al*.^27^ assessed peach firmness, TSS, and skin and flesh color parameters; Pan, *et al*.^28^ detected cold injury in peaches. However, to the best of our knowledge, no research has been reported to predict the pectin contents of peach or other fruits using spectroscopic or hyperspectral imaging techniques, let alone measuring the distribution maps of the pectin contents for peach fruit. Moreover, it should be noticed that the above studies involving hyperspectral imaging techniques were limited to the non-destructive quality prediction of fruits by measuring hyperspectral images of fruit pericarp to predict the fruit quality and to further automatically classify fruit into grades with the final purpose of increasing its commercial value^22^. Unlike these previous works, this study will visualize the spatial variation in pectins at different cross-sections inside the peach pulp, thereby yielding a maturity map index to understand the detailed quality variation of ripening and postharvest fruits. The results of this study could be used to further study the influence of planting patterns and postharvest storage regimes and optimize them.

Given the limited efforts in the investigation of visualizing the spacial distribution maps of quality attributes inside peach pulps, the main objective of this study was to investigate the potential of hyperspectral imaging for generating the distribution maps of pectin contents inside peach pulp. Specifically, three kinds of pectins, namely, protopectin, water-soluble pectin, and total pectin, were analyzed in this work.

## Results

### Spectral features of peach pulp

The spectral profiles extracted from the hyperspectral images of the peach pulp in spectral sets I (380–1030 nm) and II (874–1734 nm) are shown in Supplementary Fig. S1. The spectral profiles contain the spectral information of the main components of the peach pulp, which is important for quantitative predicting the pectin contents of peach pulp. The spectral profiles in the visible spectral region (400–700 nm) show low reflectance in the blue region (approximately 380–450 nm) and high reflectance in the red region (approximately 600–700 nm). Absorbance peaks at approximately 675 nm were found in some spectra, which was due to the presence of red-absorbing pigments, particularly chlorophyll. Absorption peaks were also found in the near-infrared region (700–1700 nm), which were the responses of the electromagnetic vibration of molecular bonds, such as those of C-H, O-H, N-H and C-O. In particular, there were three absorption peaks at approximately 760 nm (shown in Supplementary Fig. S1a), 980 nm (shown in Supplementary Fig. S1a and b), and 1460 nm (shown in Supplementary Fig. S1b), which were assigned to the third, second and first overtones of O–H stretching, respectively. Another dominant absorption band was found at approximately 1200 nm, which was assigned to the first and second overtones of C–H stretching and the combination of C–H stretching and deformation of the first overtones. However, the extracted spectral profiles covered the majority of the visible and near-infrared region, which is based on molecular overtone and combination vibrations of mid-infrared absorption features. Therefore, the visible and near-infrared bands are typically very broad, leading to complex spectra, and it is difficult to assign prominently extraordinary peaks related to pectins. Especially, when more samples were considered (as shown in Supplementary Fig. S1), their spectra become too complex to be analyzed by the direct observation of the spectral profile figures. For the analysis of visible and near-infrared spectra, instead of direct observation of the spectral profile figures, multivariate data analyses are often employed to extract the desired chemical information and establish quantitative spectral models. In this work, the quantitative spectral models were established through multivariate data analysis between the measured visible and near-infrared spectra extracted from the hyperspectral images of the cross-sections for peach pulp and their reference to pectin contents.

### Protopectin analysis

Because no featured spectral peaks related to protopectin, the full-range spectra were used to establish the calibration models to understand the overall abilities of the visible and near-infrared hyperspectral imaging technique for the prediction of the protopectin content in the peach pulp. The most important wavelengths for the protopectin prediction were then selected using four selection strategies, namely, the successive projections algorithm (SPA), uninformative variable elimination (UVE), UVE-SPA and competitive adaptive reweighted sampling (CARS), and their performances were compared to choose the best strategy.

### Calibration based on full-range spectra

Two spectral sets were analyzed, and two calibration methods of partial least squares regression (PLSR) and least squares support vector machines (LS-SVM) were used separately to establish the calibration models. When spectral set I was considered, the PLSR and LS-SVM models established using original spectral data (ori-full-PLSR-I model and ori-full-LS-SVM-I model) had good results with *r*_*val*_ values of 0.857 and 0.846, respectively. When spectral set II was considered, poorer predictions were obtained. Specifically, the values for the ori-full-PLSR-II model and for the ori-full-LS-SVM-II model were 0.781 and 0.769, respectively. When spectral preprocessing was applied, Supplementary Table S1 shows that the predictive capabilities of the models established based on both spectral set I and II were little improved, as indicated by the similar *r*_*val*_ and root mean square errors of cross-validation (RMSECV) values of the original and preprocessed models. In addition, in terms of robustness, the PLSR-based models were much more robust than the LS-SVM models. The AB_RMSE values of the PLSR-based models were only approximately 1/3 and 1/2 of those of the LS-SVM-based models for spectral sets I and II, respectively. The results of the protopectin prediction based on the full-range spectra indicate that the PLSR-based models had similar prediction accuracy but better robustness than the LS-SVM-based models, and the spectral preprocessing did not improve the prediction in terms of both accuracy and robustness.

### Model optimization by selecting important wavelengths

**Figure 1:**
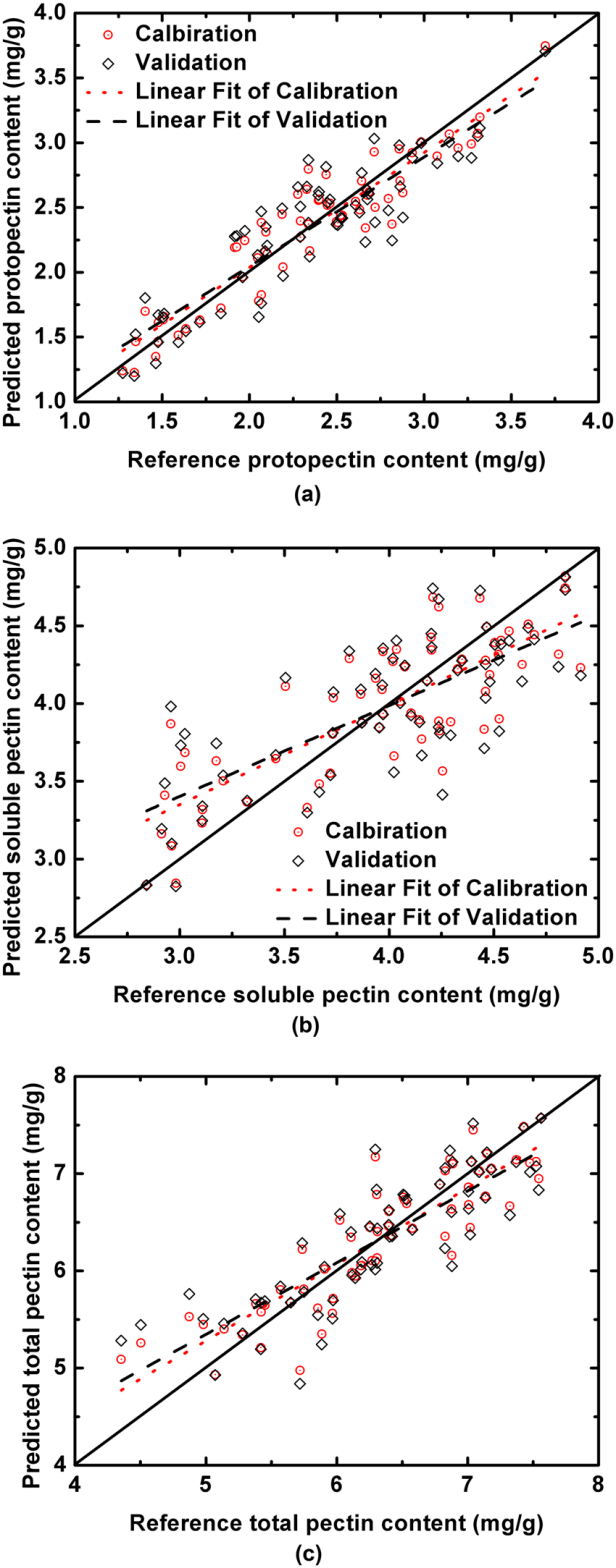
Measured versus predicted pectin contents of the peach pulp by the best models for protopectin (**a**), water-soluble pectin (**b**), and total pectin (**c**).

When the full-range spectra were used for predicting the protopectin content, an overall usefulness of the visible and near-infrared spectroscopy was given, in which the good predictions with RMSECV values lower than 0.3 were obtained. The selection of the most important variables was then considered to evaluate whether these variables could further improve the predictive ability of the hyperspectral imaging in terms of accuracy and robustness. When the SPA was considered to select the wavelengths with no multicollinearity, compared with the models established based on the full-range spectra, the SPA-based models were simpler and had slightly better predictions. Specifically, (1) the wavelength numbers were 512 and 256 for the full-spectral models based on the spectral sets I and II, respectively, whereas the mean numbers for the SPA models were 8 and 4, only 1.56% of the full-range spectral models; (2) the mean RPD of all eight SPA models (including the two spectral sets and two calibration methods with or without preprocessing) was 108.3% of all the eight corresponding full-spectral models, showing that the wavelength selection using SPA improved the prediction. When the CARS was considered, the eight CARS models (including the two spectral sets and two calibration methods, with or without preprocessing) had a similar prediction as the SPA models (mean RPD 1.902 vs. 1.850). On the other hand, the calculation of UVE and UVE-SPA strategies did not improve the predictions; their mean RPD values were 1.682 and 1.642, respectively. According to the results in Supplementary Table S1, hyperspectral imaging was an effective alternative for quantitatively determining the protopectin content of peach flesh. The highest RPD of 2.264 was obtained by the SPA-LS-SVM model without preprocessing from spectral set I. Figure 1a shows the scatter plot of the measured versus the predicted protopectin content of the best model. It was noted that the sample points are distributed closely to the regression line, which shows a good prediction of protopectin content in the peach flesh using hyperspectral imaging.

The CCD detector of a hyperspectral imaging system is the key element to quantify the intensity of the acquired light by converting incident photons into electrons. In this work, the spectra of spectral sets I and II were measured using CCD detectors, which were made of silicon (Si) and indium gallium arsenide (InGaAs), respectively. System I used a silicon-based CCD camera, which is commonly used for acquiring the spectral information in the visible and shortwave near-infrared regions, whereas system II used the InGaAs-based CCD camera, which is commonly used for detecting the spectra at 900–1700 nm^29^. These two spectral ranges are commonly used for hyperspectral imaging techniques for food quality analyses, including fruits^22^. According to the results in Supplementary Table S1, the performances of the two detectors were compared. It is clear that the Si-based models had a better prediction than the InGaAs-based models. The mean RMSECV of the Si-based models was 0.300, which was only 46% of that of the InGaAs-based models (mean RMSECV was 0.646). Therefore, it is recommended to use a hyperspectral imaging system with an Si detector to predict the protopectin content of peach fruit. At the same time, this work also considered preprocessing for the analysis of hyperspectral images. However, when comparing the corresponding models with and without preprocessing, no obvious improvement in either accuracy or robustness was found after preprocessing. This might be because the measurement surfaces of the peach flesh were flat; thus, few negative effects were generated from random noise, variation in length of the light path, and light scattering. On the other hand, the two typical PLSR and LS-SVM modeling methods were considered and compared. In general, the two methods had similar prediction among the twenty models (two spectral sets × five wavelength sets (full spectral range and the sets selected by SPA, UVE, UVE-SPA, and CARS) × with and without preprocessing). In addition, by analyzing the AB_RMSE of all of the models for the protopectin analysis, most models had good robustness with an AB_RMSE less than 0.1. Only six models had an AB_RMSE of more than 0.1; two of these models were based on spectral set I, whereas the other four models were based on spectral set II.

### Water-soluble pectin analysis

Similar to the protopectin analysis, the investigation of water-soluble pectin started with the calibration based on the full range spectra with the spectra with and without preprocessing of the two spectral sets; the results are shown in Supplementary Table S2. It was noticed that when the full spectral range was considered, spectral set I had slightly better prediction than spectral set II, for which the mean RPD values were 1.235 and 1.113, respectively. It was obvious that the prediction of the water-soluble pectin was much worse than that of the protopectin when the full-range spectra were used for the calibration. Moreover, when the wavelength selection was carried out, still little improvement occurred. The best model for the water-soluble pectin prediction was the CARS-PLSR model without preprocessing from spectral set I, which had an RPD of 1.447. The scatter plot of the best model for the water-soluble pectin prediction is shown in Fig. 1b. In contrast to the scatter plot of the protopectin prediction, many sample points in Fig. 1b are far away from the regression line, showing that the prediction of the water-soluble pectin was not strong. In addition, the Si-based models also had a better prediction than the InGaAs-based models for the water-soluble pectin prediction. The mean RMSECV values of all twenty kinds of Si- and InGaAs-based models were 0.461 and 0.683, respectively. In addition, similar to the protopectin analysis, no obvious improvement after preprocessing in either accuracy or robustness was found for the water-soluble pectin predictions, and the PLSR-based models and LS-SVM-based models had similar results.

### Total pectin analysis

In addition to the prediction of protopectin and water-soluble pectin, hyperspectral imaging was also evaluated for the prediction of the total pectin content in the peach pulp. As shown in Supplementary Table S3, the InGaAs-based models made a poor prediction based on both the full-range spectra and the important spectra at the selected wavelengths. The mean RPD of all corresponding twenty models was only 1.087. On the other hand, the prediction of the Si-based models was also not good when the full-range spectra were used for the model calibration, which had a mean RPD of 1.363 across the four models. When the four strategies for the wavelength selection were applied to select the important wavelengths from the full-range spectra, the predictive accuracy improved. The best model was the CARS-PLSR model without preprocessing from spectral set I, which had an RPD of 1.830. The robustness of the best model for the prediction of the total pectin was good, with an AB_RMSE value of 0.065. Figure 1c shows the scatter plot of the best model for the total pectin prediction. The sample points in Fig. 1c are not as close to the regression line as those in Fig. 1a but are closer than those in Fig. 1b. In addition, similar to the other two pectin predictions, preprocessing did not clearly improve the predictive accuracy for the total pectin analysis, and there were few differences between the PLSR-based models and the LS-SVM-based models.

### Visualization of pectin distribution maps for peach pulp

**Figure 2:**
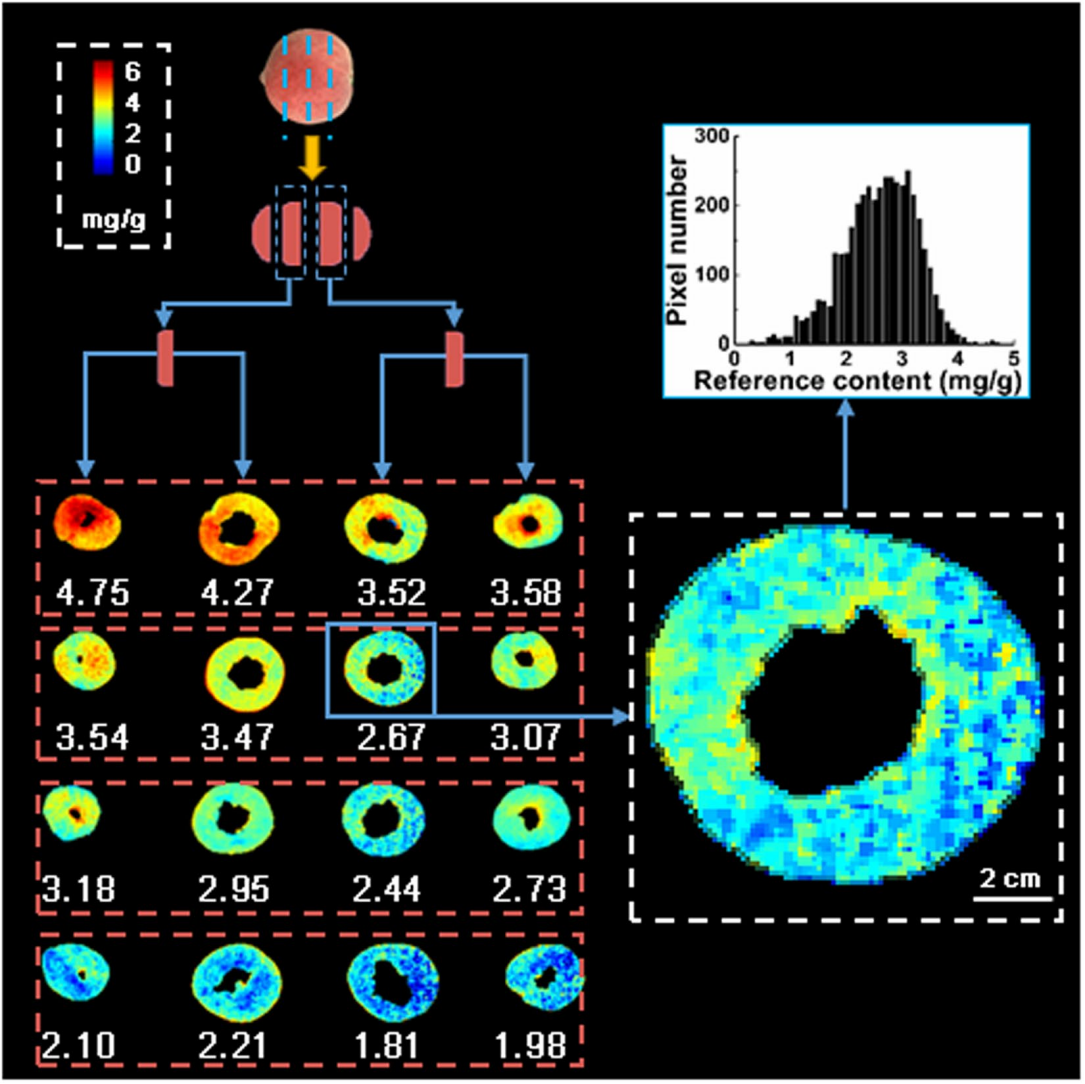
Examples of the maps of the protopectin distribution inside peach pulp from the fruits with different protopectin contents. The figure at the top right corner shows an example of the histogram of protopectin contents of the pixels within the slice, with a mean content of 2.67 mg/g.

Visualizing the pectin distribution in peach pulp in a pixel-wise manner is important to observe the detailed pectin variation in the fruit. Such visualization is the main advantage of hyperspectral imaging compared to the carbazole colorimetry method, which only measures the total content value of each kind of pectin for a certain part of the fruit pulp. In this work, the distribution maps were generated by inputting the spectrum of each pixel within the hyperspectral images of the fruit pulp into the best models to obtain the pectin contents of each pixel. Only the distribution maps of the protopectin contents were generated, as the predictions of the water-soluble pectin and the total pectin were not strong. Figure 2 shows the examples of distribution maps of some of the tested samples with different mean contents of protopectin. The four slices at each row are from one fruit. The predicted protopectin contents of all pixels were mapped with a linear color scale using different colors from red to blue (color bar in Fig. 2), representing different contents from high to low, in order to more easily clarify the content variation of protopectin in the peach pulp. In Fig. 2, the pixels with high values of approximately 6 mg/g are shown in red and those with low values of approximately 1 mg/g are shown in blue. Numbers under the distribution maps in the red boxes are the average protopectin contents for corresponding fruit slices. As shown in Fig. 2, the upper-side slices with higher average contents of protopectin are mainly shown in red and yellow colors, whereas the down-side slices with lower contents are mainly shown in the green and blue colors. Moreover, different slices had various protopectin contents even if they were from the same fruit. On the other hand, there were also a considerable range of protopectin content even within one slice, which is difficult to be observed using the carbazole colorimetry method. Take the slice with a mean content of 2.67 mg/g in the second row and third column as an example. Its large high resolution image and the histogram of protopectin contents of the pixels within this slice are shown in Fig. 2. The content range of all the pixels within this slice was from 0 to 5 mg/g, with a standard deviation of 0.67 mg/g. The results show that the results from the carbazole colorimetry method are not representative for heterogeneous materials, such as peach pulp, because the carbazole colorimetry method can only obtain a total pectin content of a pulp cube. The measured contents from several pulp cubes using the carbazole colorimetry method cannot represent the pectin conditions of the whole fruit. However, such differences in the protopectin contents from different locations within the same fruit could be easily discerned using the hyperspectral imaging technique. In addition, it should be noted that because of the limited penetration depths of hyperspectral imaging of peach flesh, the generated distribution maps can only show the protopectin contents across the exposed surface of the fruit slice, rather than the regions of the fruit that are 1.5 cm apart (the thickness of a section).

## Discussion

The results in Supplementary Table S1 and Fig. 2 confirmed the potential of hyperspectral imaging to provide detailed distribution of protopectin contents at the pixel level inside peach pulp. The predictions of the water-soluble pectin and the total pectin were not poor. This might be because the slicing of a fruit to create the scanning surface might lead to the spreading of water-soluble pectins across the surface of the fruit section to be imaged, resulting in a poor prediction for water-soluble pectin by the hyperspectral imaging technique. Since the content of total pectin is the sum of protopectin and water-soluble pectin, the prediction for the total pectin was also affected. However, because the peach pericarp has very strong absorption of spectra in the visible and near-infrared regions, the fruit flesh of unsliced fruit is not measurable by hyperspectral imaging. The visualization of water-soluble pectin and the total pectin contents of pulp for intact fruits will be considered in future works. Moreover, in this work, quantitative spectral models were established through multivariate data analysis between the measured visible and near-infrared spectra extracted from the hyperspectral images of the cross-sections of peach pulp and their reference pectin contents, and the results showed that the model for protopectin analysis had good correlation and robustness between the spectral data and the reference values.. Therefore, the established quantitative spectral model and the generated distribution maps in Fig. 2 are reliable and can only be used to predict protopectin, water-soluble pectin, and total pectin contents. Nevertheless, it should be noted that this is a preliminary work focusing on the establishment of the models. In future works, more samples from different varieties, different climate and soil conditions, different preharvest planting patterns and postharvest storage strategies, different ripening stages, and different years will be considered to further improve the applicability and robustness of the models, which will be used to generate pectin distributions. The obtained distribution maps will then be used to describe peach ripening-related changes in pectins and will then be compared with the chemically defined peach pectins at different developmental stages. In addition, for the changes of the pectin content during ripening, whether the pectins are homogalacturonans or pectins with complex, neutral sugar-rich side chains or pectins that are being cleaved are also important. To predict more detailed pectin polymer structures, such as the branched or neutral sugar-rich pectins or the Me-esterified or unesterified homogalacturonan pectins, other specific models should be established for these pectins, which will be considered in future works. Another interesting work that will be considered in future is to investigate the presence and distribution of polygalacturonase for different kinds of peaches (clingstone vs. freestone) ripening to a different final texture (“melting” vs “more crisp/firm”).

The hyperspectral imaging technique used in this work generated pectin distribution maps across the whole surface of the fruit sections with the advantages of low cost, high imaging speed and minimal sample preparation. On the other hand, immunochemistry techniques such as those involving monoclonal antibodies have inherent advantages of high sensitivity and great specificity to the labeled molecule. In future works, immunochemistry techniques will be coupled to hyperspectral imaging to give a more comprehensive analysis of the different type of pectins for peach flesh. Moreover, it is also important to explore the pectic heterogeneity within cell walls at the cellular level. In future works, immunohistochemistry techniques can also be used to investigate the changes of microstructural properties at the cellular level throughout peach fruit softening, which is an important expansion of the present study. In addition, in this study, the sulfuric acid-carbazole colorimetry method, which is the traditional and commonly used method for pectin measurement of fruits and vegetables^18–21^, was used to measure the reference pectin contents. However, the carbazole assay can give a positive signal with some wall pentoses (e.g., Ara) that are often components of neutral sugar side chains of some complex pectins. In future works, m-phenylphenol method^30^ will also be considered for the determination of uronic acids for the model calibration.

This is the first work using hyperspectral imaging techniques to predict the pectin contents of fruit pulp and generate distribution maps of the pectins that are inside the fruits. To the best of our knowledge, the pectin contents of fruits have not been predicted by either visible and near-infrared spectroscopy or hyperspectral imaging techniques. This might be because the pectins related to the firmness of the fruits mainly exist in the fruit pulp, whereas the majority of works involving spectroscopy and hyperspectral imaging techniques for the quality prediction of fruits are based on spectral measurements of the fruit pericarp. Although the pectin distribution cannot be quantitatively observed by the visual inspection or the carbazole colorimetry method, it can be visualized at the pixel level within peach pulp by analyzing hyperspectral images. Such pectin distribution maps could be used as an indicator to provide detailed information for understanding the ripening and postharvest processes of the fruits and could be used to further optimize the preharvest planting patterns and postharvest storage regimes. As the first research on predicting and visualizing the pectin contents of peach pulp at the pixel level, it opens up an attractive prospect of using the proposed hyperspectral-imaging-based method to further investigate the softening process of other fruits during the preharvest and postharvest periods, and this research will promote more efforts in investigating hyperspectral imaging for quantitatively mapping constituents of interest in complex food matrices at the pixel level.

## Methods

**Figure 3:**
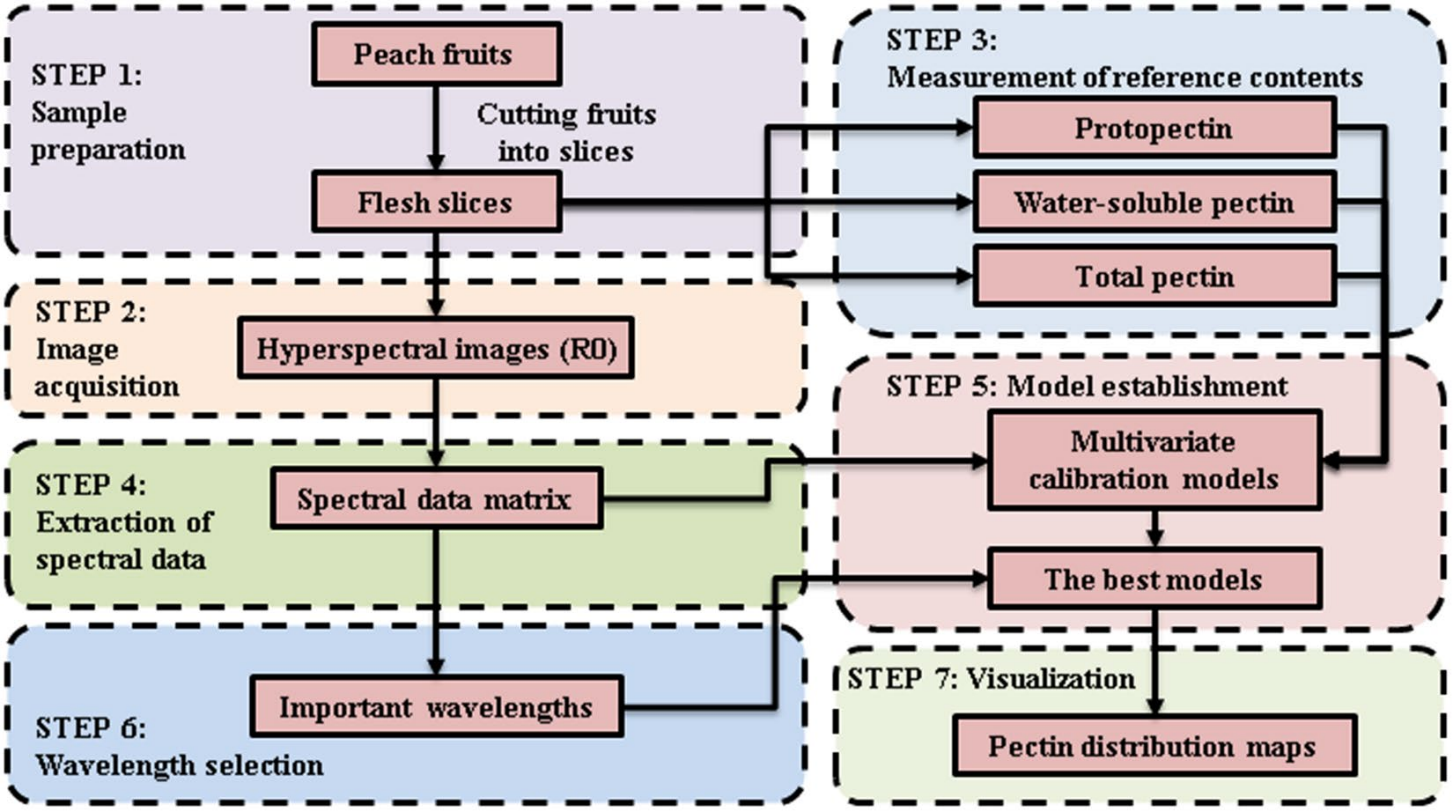
Key steps for the whole procedure.

The key steps for the whole experiments are shown in Fig. 3. The main works of this study included (1) sample preparation and acquisition of the hyperspectral images at different cross-sections inside peach pulp (steps 1 and 2 in Fig. 3, described in the sections 4.1 and 4.2); (2) measurement of the reference pectin contents of samples (step 3 in Fig. 3, described in the section 4.3) and extraction of the spectral data of samples from their hyperspectral images (step 4 in Fig. 3, described in the section 4.4); (3) establishment of multivariate calibration models between the extracted spectra of the samples and their reference pectin contents using PLSR and LS-SVM, respectively (step 5 in Fig. 3, described in the section 4.5); (4) selection of the most important wavelengths by using three selection algorithms of SPA, UVE, and CARS to improve the accuracy and robustness of the prediction (step 6 in Fig. 3, described in the section 4.6); and (5) visualization of the pectin distribution maps at the pixel level at different cross-sections inside peach pulp based on the best models (step 7 in Fig. 3, described in the section 4.7).

### Sample preparation

Peach (*Prunus persica* L. Batsch cv. Hujingmilu) fruits were harvested were harvested from a commercial orchard in Jiaxing, Zhejiang Province, in 2015. The fruits were transported to the laboratory on the day of harvest, and those of uniform commercial maturity with absence of disease and mechanical wounding were selected. To obtain reasonable ranges of pectins to establish robust spectral calibration models, the fruits were divided into two batches and then stored at 20 °C or 8 °C. Specifically, there were four sampling points, including 2, 4, and 8 days at 20 °C and 8 days at 8 °C after the start of the experiment. Thirty-two fruits were sampled at each sampling point. As a result, there were 128 peach fruits prepared for further analysis.

### Image acquisition

To obtain the pectin distributions inside peach pulp, each fruit was cut into two slices with a thickness of 1.5 cm, resulting in four images obtained for each fruit (Fig. 2). Three cross-sections were taken from both slices, all parallel to the equatorial axis of the fruit. Hyperspectral images of the cross-sections were acquired by two hyperspectral imaging systems in reflectance mode at 380–1030 nm (measured by system I) and 874–1734 nm (measured by system II). Each system consists an imaging spectrograph, a high-resolution camera, and a camera lens, and two systems shared dual 150-W quartz tungsten halogen lamps as the light source and a conveyer belt operated by a stepper motor. The illumination and image acquisition were performed on the same side of the sample, where the illumination was focused on an area adjacent and parallel to the detector’s field of view. The details of the systems and the schematic diagram of their main components are described in the literature^31^. During the image acquisition, each slice sample was placed on the conveyer belt to be scanned line by line to build a hyperspectral image called “hypercube” with dimensions of (*x, y*, and *λ*). For each scan, a two-dimensional image (*y, λ*) with the whole spectral dimension (*λ*) and with one spatial dimension (*y*) was acquired. A complete hyperspectral cube was taken as the line was scanned along the direction of *x* dimension and was stored in band-interleaved-by-line format. Among the 128 total fruits prepared, half were used for the analysis of visible and shortwave-near infrared spectra (380–1030 nm, spectral set I) measured by system I, and the remaining 64 fruits were used for the analysis of longwave near-infrared spectra (874–1734 nm, spectral set II) measured by system II. After the image acquisition, the raw hyperspectral images were corrected into reflectance images for further analysis using the method described in the literature^32^.

### Measurement of reference pectins

Hyperspectral imaging is an indirect method, which needs reference values of the interested attributes to be measured for model calibration with the spectral data. In this study, the reference contents of protopectin, water-soluble pectin, and total pectin of the fruit samples were determined immediately after the acquisition of hyperspectral images using the sulfuric acid-carbazole colorimetry method^21^. A 1.0-mL solution was mixed with 0.25 mL of 0.1% carbazole-ethanol solution, and 5.0 mL of sulfuric acid was quickly added within 6 s. The mixed solution was incubated at 85 °C for 20 min and cooled quickly in a water bath, after which the absorbance at 525 nm (A525 nm) was measured. All experiments were performed in triplicate. It should be noted that the reference pectin contents measured by the sulfuric acid-carbazole colorimetry method were expressed as galacturonic acid equivalents, and do not reflect the branched rhamnogalacturonan pectins with extensive neutral sugar substituents. Therefore, the protopectin measured by the sulfuric acid-carbazole colorimetry method was only homogalacturonan and expressed as galacturonic acid equivalents. The measured water-soluble pectin was also homogalacturonan and expressed as galacturonic acid equivalents, and total pectin is a sum of measured protopectin and water-soluble pectin. After measurement, the reference values were then used for the model calibration by establishing their quantitative relationship with the spectral profiles of the fruit samples. For each fruit, only the reference pectin contents of the slice closed to the fruit stem were measured and further used to establish quantitative spectral models. Supplementary Table S4 summarizes the variations in the three pectin indices of the peach samples. Wide variations were obtained for the three pectin indices, which was important for establishing accurate and robust calibration models.

### Spectral extraction and preprocessing

Spectral data extraction was an important step to establish a quantitative spectral model. The establishment of the quantitative models requires that the regions for extracting the spectra be from the sample whose pectin contents were measured by the carbazole colorimetry method. Otherwise, such establishment is meaningless. In this study, there were two ways to guarantee the representative measured spectra. First, only the spectra from the pixels of the fruit slice were extracted from the hyperspectral images, while those from the background and fruit pericarp were ignored. Second, all pixels of both the front and back of the cross-sections of the fruit slice were selected, making the majority of the area of the pulp cross-sections included. The pixel selection was carried out manually using the region of interest (ROI) function of ENVI v4.6 software (Research Systems Inc., Boulder, CO, USA). After the pixel selection, the spectra of all pixels within the identified ROI of the sample were extracted. A mean spectrum of the ROI was then calculated based on the spectra of all pixels within this ROI and was set as the characteristic spectrum of the slice sample to which the ROI belonged. To correct the effects from random noise, variation due to light path length, and light scattering, spectral preprocessing is usually performed mathmatically prior to the model calibration^29^. In this study, two commonly used preprocessing algorithms called Savitzky–Golay smoothing and standard normal variate transformation were considered.

### Model calibration and evaluation

The hyperspectral imaging technique used in this works covered the visible and near-infrared regions, which are based on molecular overtone and combination vibrations of mid-infrared absorption features. Therefore, the visible and near-infrared bands are typically very broad (as shown in Supplementary Fig. S1), leading to complex spectra. Because of these broad bands, it is difficult to assign specific spectral features to specific chemical components. Multivariate data analyses are often used to extract the desired chemical information and establish quantitative spectral models. In this work, on the basis of the measured hyperspectral images of the cross-sections for peach pulp and their reference pectin contents, quantitative spectral models were established through multivariate data analysis and used for the further pectin prediction of each pixel and the generation of pectin distribution maps. Partial least squares regression (PLSR)^33^ and least squares support vector machines (LS-SVM)^34^ were considered and compared in this study. Full leave-one-out cross-validation was used to evaluate the established spectral calibration models. Within the processes of calibration and validation, the performance of a calibration model is evaluated in terms of its root mean square error of calibration (RMSEC) and correlation coefficient of calibration (*r*_*val*_) in the calibration process, as well as its RMSECV, correlation coefficient of cross-validation (*r*_*val*_), and residual predictive deviation (RPD) in the cross-validation process. In addition, the absolute difference between the RMSEC and RMSECV (AB_RMSE) is used for the evaluation of model robustness. A small AB_RMSE indicates that a model is robust, whereas a large one indicates overfitting. In general, a good model should have higher *r*_*val*_, *r*_*val*_, and RPD values, and lower RMSEC, RMSECV and AB_RMSE values.

### Model optimization

Hyperspectral images suffer from a great degree of dimensionality, with redundancy among contiguous variables (wavelengths); the selection of only important and necessary wavelengths can avoid the colinearity among the wavelengths, resulting in more optimized calibration models^35^. In this work, SPA^36^, UVE^37^, and CARS^38^ were used to select the most important variables that had less redundancy but that contributed the most to the pectin measurement of peach pulp.

### Visualization of pectin distribution maps

The visualization of pectin spatial distribution is important for observing the maturity variation from position to position inside peach pulp. By using hyperspectral imaging techniques in tandem with chemometrics, distribution maps of pectins of pulp cross-sections were generated based on the spatial position of every pixel and the corresponding spectral values. After comparing calibration models established using different spectral sets and calibration methods, only the best models with both good predictive accuracy and robustness were chosen and applied in a pixel-wise manner in the hyperspectral images of peach pulp to produce the distribution maps. There were two steps of visual representation. The pectin content of each pixel was first calculated by inputting its spectrum into the established quantitative model. Such calculation was repeated for all pixels of peach pulp to obtain their pectin contents. A distribution map of the pectins within the pulp cross-section was then generated according to the spatial position of every pixel and their predicted pectin contents. To enhance the contrast of distribution maps and make the distribution maps easily interpretable for discovering differences in pectin contents inside the peach pulp, a linear color scale (color bar in Fig. 2) was used to map the predicted values of every pixel into different colors (the pixels with high values are shown in red and those with low values are shown in blue), resulting in a pseudo-color map. All of the computations, chemometric analyses and visualization process were performed using ENVI v4.6 (Research System, Inc., Boulder, CO, USA), “The Unscrambler v9.7” (CAMO PROCESS AS, Oslo, Norway), and programs developed by the authors in the MATLAB 2011a software (The Mathworks, Inc., Natick, MA, USA).

## Electronic supplementary material

Supplementary Information
