# Supplementary material for: Quantitative visualization of pectin distribution maps of peach fruits
Source: Sci Rep. 2017 Aug 24;7:9275. doi: 10.1038/s41598-017-09817-7 (PMC5571203; doi:10.1038/s41598-017-09817-7)

Quantitative visualization of pectin distribution maps of peach fruits

Nan Zhu[1], Weinan Huang[1], Di Wu[1]*, Kunsong Chen[1], Yong He[2]

[1] College of Agriculture & Biotechnology/ Zhejiang Provincial Key Laboratory of Horticultural Plant Integrative Biology/The State Agriculture Ministry Laboratory of Horticultural Plant Growth, Development and Quality Improvement, Zhejiang University, Zijingang Campus, Hangzhou 310058, P. R. China

[2] College of Biosystems Engineering and Food Science, Zhejiang University, Hangzhou 310058, China

* Corresponding author. Tel: +85 571 88982226. Fax: +86 571-88982224. E-mail: di_wu@zju.edu.cn, china.di.wu@gmail.com

Supplementary Table S1 Predictive results of protopectin content of peach flesh slice by using visible and near infrared hyperspectral imaging

Spectral Set	Spectral preprocessing	Wavelength selection	Variable number	Calibration	LVs	Calibration			Cross-validation				AB_RMSE
						r_{cal}	R^2_{cal}	RMSEC	r_{val}	R^2_{val}	RMSECV	RPD	
I	No	/	512	PLSR	3	0.879	0.772	0.266	0.857	0.742	0.288	1.937	0.022
I	No	/	512	LS-SVM	/	0.923	0.849	0.217	0.846	0.715	0.298	1.874	0.081
I	Yes	/	512	PLSR	3	0.891	0.794	0.254	0.868	0.760	0.277	2.011	0.024
I	Yes	/	512	LS-SVM	/	0.889	0.788	0.257	0.807	0.651	0.330	1.692	0.073
I	No	SPA	14	PLSR	10	0.941	0.885	0.189	0.898	0.811	0.246	2.264	0.057
I	No	SPA	14	LS-SVM	/	0.950	0.901	0.175	0.881	0.774	0.265	2.103	0.090
I	Yes	SPA	2	PLSR	2	0.877	0.769	0.268	0.864	0.754	0.281	1.988	0.013
I	Yes	SPA	2	LS-SVM	/	0.884	0.781	0.261	0.862	0.743	0.283	1.971	0.022
I	No	UVE	157	PLSR	7	0.908	0.825	0.234	0.874	0.770	0.272	2.055	0.038
I	No	UVE	157	LS-SVM	/	0.910	0.827	0.232	0.848	0.718	0.296	1.882	0.064
I	Yes	UVE	14	PLSR	7	0.875	0.765	0.271	0.822	0.683	0.319	1.749	0.048
I	Yes	UVE	14	LS-SVM	/	0.925	0.842	0.222	0.7381	0.542	0.377	1.478	0.155
I	No	UVE-SPA	6	PLSR	5	0.882	0.779	0.263	0.856	0.739	0.289	1.929	0.027
I	No	UVE-SPA	6	LS-SVM	/	0.923	0.850	0.216	0.853	0.727	0.292	1.913	0.076
I	Yes	UVE-SPA	5	PLSR	4	0.836	0.699	0.306	0.790	0.633	0.343	1.626	0.037
I	Yes	UVE-SPA	5	LS-SVM	/	0.839	0.700	0.306	0.661	0.424	0.423	1.319	0.117
I	No	CARS	4	PLSR	4	0.883	0.780	0.262	0.868	0.762	0.277	2.016	0.015
I	No	CARS	4	LS-SVM	/	0.887	0.786	0.258	0.872	0.760	0.273	2.042	0.015
I	Yes	CARS	8	PLSR	1	0.877	0.769	0.268	0.867	0.759	0.278	2.005	0.010
I	Yes	CARS	8	LS-SVM	/	0.882	0.778	0.263	0.8559	0.732	0.289	1.932	0.026

II	No	/	256	PLSR	5	0.837	0.701	0.568	0.781	0.619	0.651	1.595	0.083
II	No	/	256	LS-SVM	/	0.859	0.736	0.534	0.769	0.589	0.666	1.561	0.132
II	Yes	/	256	PLSR	4	0.833	0.694	0.575	0.781	0.619	0.652	1.594	0.077
II	Yes	/	256	LS-SVM	/	0.845	0.711	0.559	0.710	0.489	0.743	1.399	0.184
II	No	SPA	5	PLSR	4	0.840	0.706	0.564	0.796	0.643	0.631	1.647	0.067
II	No	SPA	5	LS-SVM	/	0.841	0.707	0.563	0.796	0.632	0.630	1.649	0.067
II	Yes	SPA	3	PLSR	2	0.816	0.666	0.601	0.790	0.635	0.637	1.630	0.036
II	Yes	SPA	3	LS-SVM	/	0.826	0.680	0.588	0.763	0.582	0.672	1.547	0.084
II	No	UVE	88	PLSR	4	0.828	0.685	0.583	0.785	0.624	0.647	1.606	0.064
II	No	UVE	88	LS-SVM	/	0.852	0.724	0.546	0.781	0.607	0.652	1.594	0.106
II	Yes	UVE	40	PLSR	4	0.807	0.651	0.614	0.768	0.600	0.668	1.557	0.054
II	Yes	UVE	40	LS-SVM	/	0.831	0.689	0.580	0.760	0.576	0.676	1.537	0.097
II	No	UVE-SPA	3	PLSR	2	0.807	0.652	0.613	0.781	0.621	0.649	1.600	0.036
II	No	UVE-SPA	3	LS-SVM	/	0.827	0.683	0.585	0.761	0.575	0.677	1.534	0.092
II	Yes	UVE-SPA	5	PLSR	4	0.825	0.681	0.587	0.788	0.630	0.642	1.620	0.055
II	Yes	UVE-SPA	5	LS-SVM	/	0.831	0.691	0.578	0.734	0.523	0.718	1.450	0.140
II	No	CARS	9	PLSR	4	0.862	0.742	0.527	0.828	0.694	0.584	1.780	0.057
II	No	CARS	9	LS-SVM	/	0.866	0.749	0.520	0.823	0.677	0.590	1.760	0.070
II	Yes	CARS	8	PLSR	5	0.874	0.763	0.505	0.845	0.722	0.557	1.866	0.052
II	Yes	CARS	8	LS-SVM	/	0.871	0.759	0.510	0.835	0.697	0.572	1.818	0.062

LVs: Number of latent variables

Supplementary Table S2 Predictive results of soluble pectin content of peach flesh slice by using visible and near infrared hyperspectral imaging

Spectral Set	Spectral preprocessing	Wavelength selection	Variable number	Calibration	LVs	Calibration			Cross-validation				AB_RMSE
						r_{cal}	R^2_{cal}	RMSEC	r_{val}	R^2_{val}	RMSECV	RPD	
I	No	/	512	PLSR	8	0.733	0.537	0.386	0.566	0.307	0.480	1.183	0.094
I	No	/	512	LS-SVM	/	0.886	0.765	0.275	0.572	0.298	0.476	1.193	0.200
I	Yes	/	512	PLSR	4	0.663	0.440	0.425	0.568	0.333	0.471	1.206	0.046
I	Yes	/	512	LS-SVM	/	0.939	0.872	0.203	0.555	0.225	0.500	1.136	0.296
I	No	SPA	4	PLSR	3	0.660	0.435	0.435	0.564	0.327	0.473	1.200	0.038
I	No	SPA	4	LS-SVM	/	0.898	0.789	0.260	0.659	0.427	0.430	1.321	0.169
I	Yes	SPA	3	PLSR	2	0.624	0.390	0.443	0.557	0.327	0.473	1.200	0.030
I	Yes	SPA	3	LS-SVM	/	0.663	0.436	0.426	0.561	0.313	0.470	1.206	0.044
I	No	UVE	54	PLSR	8	0.729	0.531	0.388	0.560	0.302	0.481	1.179	0.093
I	No	UVE	54	LS-SVM	/	0.813	0.646	0.337	0.521	0.252	0.491	1.156	0.153
I	Yes	UVE	209	PLSR	4	0.660	0.436	0.426	0.575	0.344	0.467	1.215	0.041
I	Yes	UVE	209	LS-SVM	/	0.782	0.603	0.357	0.529	0.254	0.490	1.158	0.133
I	No	UVE-SPA	8	PLSR	7	0.783	0.614	0.353	0.664	0.438	0.432	1.314	0.079
I	No	UVE-SPA	8	LS-SVM	/	0.859	0.734	0.293	0.565	0.261	0.488	1.164	0.195
I	Yes	UVE-SPA	5	PLSR	4	0.661	0.437	0.426	0.585	0.357	0.462	1.228	0.036
I	Yes	UVE-SPA	5	LS-SVM	/	0.701	0.489	0.406	0.5585	0.298	0.475	1.194	0.070
I	No	CARS	8	PLSR	7	0.800	0.641	0.340	0.727	0.537	0.392	1.447	0.052
I	No	CARS	8	LS-SVM	/	0.891	0.789	0.261	0.674	0.442	0.424	1.339	0.163
I	Yes	CARS	8	PLSR	3	0.719	0.517	0.395	0.682	0.477	0.417	1.362	0.022
I	Yes	CARS	8	LS-SVM	/	0.721	0.517	0.394	0.642	0.412	0.435	1.304	0.041

II	No	/	256	PLSR	1	0.480	0.230	0.676	0.423	0.200	0.700	1.101	0.024
II	No	/	256	LS-SVM	/	0.567	0.300	0.645	0.317	0.062	0.747	1.033	0.101
II	Yes	/	256	PLSR	4	0.641	0.411	0.592	0.496	0.250	0.678	1.137	0.086
II	Yes	/	256	LS-SVM	/	0.650	0.409	0.593	0.432	0.177	0.699	1.103	0.107
II	No	SPA	9	PLSR	8	0.701	0.491	0.550	0.571	0.330	0.641	1.203	0.091
II	No	SPA	9	LS-SVM	/	0.732	0.534	0.526	0.413	0.100	0.731	1.054	0.205
II	Yes	SPA	4	PLSR	3	0.628	0.395	0.600	0.549	0.314	0.649	1.189	0.049
II	Yes	SPA	4	LS-SVM	/	0.643	0.411	0.592	0.445	0.174	0.701	1.101	0.109
II	No	UVE	69	PLSR	5	0.552	0.304	0.643	0.464	0.231	0.687	1.122	0.044
II	No	UVE	69	LS-SVM	/	0.693	0.473	0.559	0.406	0.157	0.708	1.089	0.148
II	Yes	UVE	68	PLSR	4	0.644	0.415	0.590	0.508	0.264	0.672	1.148	0.082
II	Yes	UVE	68	LS-SVM	/	0.709	0.491	0.550	0.484	0.220	0.681	1.133	0.131
II	No	UVE-SPA	8	PLSR	3	0.665	0.442	0.576	0.581	0.350	0.631	1.222	0.055
II	No	UVE-SPA	8	LS-SVM	/	0.687	0.470	0.561	0.453	0.165	0.705	1.094	0.143
II	Yes	UVE-SPA	5	PLSR	3	0.616	0.380	0.607	0.541	0.308	0.651	1.184	0.044
II	Yes	UVE-SPA	5	LS-SVM	/	0.629	0.393	0.601	0.504	0.251	0.667	1.156	0.066
II	No	CARS	102	PLSR	1	0.537	0.288	0.650	0.447	0.216	0.693	1.112	0.043
II	No	CARS	102	LS-SVM	/	0.565	0.304	0.643	0.437	0.191	0.693	1.112	0.050
II	Yes	CARS	3	PLSR	2	0.614	0.377	0.608	0.547	0.316	0.647	1.191	0.039
II	Yes	CARS	3	LS-SVM	/	0.676	0.456	0.569	0.474	0.206	0.687	1.123	0.118

LVs: Number of latent variables

Supplementary Table S3 Predictive results of total pectin content of peach flesh slice by using visible and near infrared hyperspectral imaging

Spectral Set	Spectral preprocessing	Wavelength selection	Variable number	Calibration	LVs	Calibration			Cross-validation				AB_RMSE
						r_{cal}	R^2_{cal}	RMSEC	r_{val}	R^2_{val}	RMSECV	RPD	
I	No	/	512	PLSR	9	0.832	0.693	0.424	0.709	0.503	0.547	1.396	0.124
I	No	/	512	LS-SVM	/	0.888	0.776	0.361	0.571	0.283	0.647	1.181	0.286
I	Yes	/	512	PLSR	7	0.820	0.672	0.437	0.725	0.531	0.531	1.438	0.094
I	Yes	/	512	LS-SVM	/	0.930	0.861	0.285	0.729	0.514	0.532	1.435	0.247
I	No	SPA	11	PLSR	10	0.870	0.756	0.377	0.806	0.654	0.456	1.674	0.079
I	No	SPA	11	LS-SVM	/	0.890	0.788	0.351	0.744	0.541	0.518	1.485	0.166
I	Yes	SPA	11	PLSR	10	0.874	0.764	0.371	0.803	0.650	0.459	1.663	0.088
I	Yes	SPA	11	LS-SVM	/	0.876	0.762	0.373	0.7801	0.606	0.48	1.594	0.107
I	No	UVE	97	PLSR	5	0.773	0.597	0.485	0.710	0.512	0.542	1.410	0.057
I	No	UVE	97	LS-SVM	/	0.783	0.610	0.477	0.686	0.468	0.557	1.371	0.080
I	Yes	UVE	188	PLSR	4	0.754	0.568	0.502	0.701	0.504	0.546	1.398	0.044
I	Yes	UVE	188	LS-SVM	/	0.916	0.836	0.309	0.7544	0.564	0.504	1.515	0.195
I	No	UVE-SPA	6	PLSR	7	0.822	0.676	0.435	0.737	0.550	0.521	1.467	0.086
I	No	UVE-SPA	6	LS-SVM	/	0.822	0.674	0.436	0.695	0.476	0.553	1.381	0.117
I	Yes	UVE-SPA	11	PLSR	10	0.876	0.768	0.368	0.810	0.662	0.451	1.694	0.083
I	Yes	UVE-SPA	11	LS-SVM	/	0.887	0.786	0.354	0.8015	0.641	0.458	1.669	0.104
I	No	CARS	11	PLSR	7	0.887	0.786	0.353	0.839	0.711	0.418	1.830	0.065
I	No	CARS	11	LS-SVM	/	0.886	0.781	0.358	0.719	0.497	0.542	1.410	0.184
I	Yes	CARS	14	PLSR	4	0.835	0.698	0.42	0.81	0.661	0.452	1.694	0.032
I	Yes	CARS	14	LS-SVM	/	0.846	0.709	0.412	0.7373	0.539	0.519	1.475	0.107

II	No	/	256	PLSR	2	0.454	0.206	0.729	0.358	0.144	0.769	1.064	0.040
II	No	/	256	LS-SVM	/	0.551	0.298	0.686	0.156	-0.078	0.850	0.964	0.164
II	Yes	/	256	PLSR	1	0.397	0.157	0.751	0.321	0.125	0.778	1.052	0.027
II	Yes	/	256	LS-SVM	/	0.548	0.287	0.691	0.360	0.122	0.767	1.067	0.076
II	No	SPA	11	PLSR	10	0.724	0.524	0.565	0.584	0.338	0.676	1.210	0.111
II	No	SPA	11	LS-SVM	/	0.723	0.517	0.569	0.450	0.156	0.752	1.090	0.183
II	Yes	SPA	4	PLSR	3	0.585	0.342	0.664	0.473	0.232	0.728	1.124	0.064
II	Yes	SPA	4	LS-SVM	/	0.609	0.370	0.650	0.451	0.181	0.741	1.105	0.091
II	No	UVE	195	PLSR	2	0.458	0.210	0.728	0.355	0.139	0.771	1.061	0.043
II	No	UVE	195	LS-SVM	/	0.555	0.299	0.685	0.223	-0.032	0.831	0.986	0.146
II	Yes	UVE	17	PLSR	4	0.500	0.250	0.709	0.428	0.204	0.742	1.103	0.033
II	Yes	UVE	17	LS-SVM	/	0.549	0.300	0.685	0.404	0.152	0.754	1.086	0.069
II	No	UVE-SPA	11	PLSR	6	0.674	0.455	0.604	0.575	0.330	0.680	1.203	0.076
II	No	UVE-SPA	11	LS-SVM	/	0.743	0.535	0.558	0.564	0.295	0.687	1.191	0.129
II	Yes	UVE-SPA	6	PLSR	5	0.631	0.398	0.635	0.512	0.269	0.711	1.151	0.076
II	Yes	UVE-SPA	6	LS-SVM	/	0.617	0.379	0.645	0.426	0.157	0.751	1.089	0.107
II	No	CARS	2	PLSR	1	0.468	0.219	0.723	0.412	0.192	0.747	1.095	0.024
II	No	CARS	2	LS-SVM	/	0.480	0.227	0.719	0.297	0.044	0.800	1.023	0.081
II	Yes	CARS	3	PLSR	2	0.496	0.246	0.711	0.406	0.178	0.754	1.086	0.043
II	Yes	CARS	3	LS-SVM	/	0.572	0.326	0.672	0.284	-0.016	0.825	0.995	0.153

LVs: Number of latent variables

Supplementary Table S4 Reference contents of protopectin, water-soluble pectin, and total pectin for peach pulp measured by the sulfuric acid-carbazole colorimetry method

Content	Maximum	Minimum	Mean	Standard deviation	Range
Protopectin	4.92	2.84	3.98	0.57	2.07
Water-soluble pectin	3.70	1.27	2.34	0.56	2.42
Total pectin	7.56	4.35	6.31	0.77	3.21

Supplementary Figure S1 Spectral profiles extracted from the hyperspectral images of the peach pulp in the spectral sets I (a) and II (b)

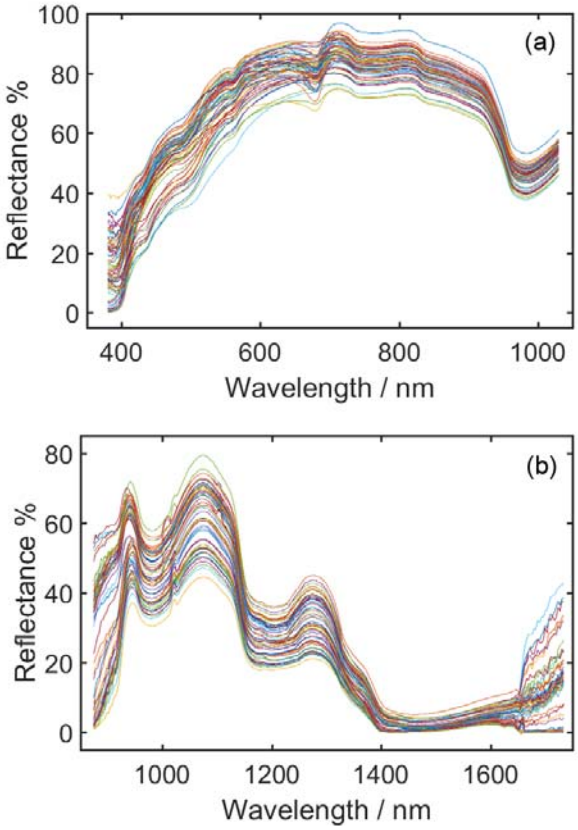

Supplement: Supplementary file 1 — Supplementary Information [file 41598_2017_9817_MOESM1_ESM.pdf]
